# Impact of Carbon Nano-Onions on *Hydra vulgaris* as a Model Organism for Nanoecotoxicology

**DOI:** 10.3390/nano5031331

**Published:** 2015-08-13

**Authors:** Valentina Marchesano, Alfredo Ambrosone, Juergen Bartelmess, Federica Strisciante, Angela Tino, Luis Echegoyen, Claudia Tortiglione, Silvia Giordani

**Affiliations:** 1 Nano-Biomolecular Group, Istituto di Scienze Applicate e Sistemi Intelligenti “E.Caianiello”, Consiglio Nazionale delle Ricerche, Via Campi Flegrei 34, 80078 Pozzuoli, Italy; E-Mails: v.marchesano@isasi.cnr.it (V.M.); a.ambrosone@isasi.cnr.it (A.A.); federica89s@hotmail.it (F.S.); angela.tino@cnr.it (A.T.); 2 Nano Carbon Materials Lab, Istituto Italiano di Tecnologia (IIT), Via Morego 30, 16163 Genova, Italy; E-Mail: juergen.bartelmess@iit.it; 3 Department of Chemistry, University of Texas at El Paso, El Paso, TX 79968, USA; E-Mail: echegoyen@utep.edu

**Keywords:** nanotoxicology, aquatic organism, *Hydra*, ecotoxicology, carbon nanomaterials, fullerenes

## Abstract

The toxicological effects of pristine and chemically modified carbon nano-onions (CNOs) on the development of the freshwater polyp *Hydra vulgaris* were investigated in order to elucidate the ecotoxicological effects of CNOs. Chemical modifications of the CNOs were accomplished by surface functionalization with benzoic acid, pyridine and pyridinium moieties. thermogravimetric analysis (TGA), Fourier transform infrared spectroscopy (FT-IR) and Raman spectroscopy confirmed the covalent surface functionalization of CNOs. *Hydra* specimens were exposed to the carbon nanomaterials by prolonged incubation within their medium. Uptake was monitored by optical microscopy, and the toxicological effects of the CNOs on *Hydra* behavior, morphology, as well as the long-term effects on the development and reproductive capability were examined. The obtained data revealed the absence of adverse effects of CNOs (in the range 0.05–0.1 mg/L) *in vivo* at the whole animal level. Together with previously performed *in vitro* toxicological analyses, our findings indicate the biosafety of CNOs and the feasibility of employing them as materials for biomedical applications.

## 1. Introduction

A large number of new carbon nanomaterials have been discovered over the last few decades [1] including fullerenes [2] multishell fullerenes, also known as carbon nano-onions (CNOs) [3], carbon nanotubes (CNTs) [4,5,6], carbon nanohorns [7], and as graphene and its derivatives [8]. Among these, CNTs and graphene have already received enormous attention from industry and the wider scientific community due to the many potential applications that are currently under investigation and development [9,10,11,12,13,14,15]. Much less attention has been given to CNOs so far, although several ways in which the unique properties of this class of compounds may be utilized have been discussed [16]. CNOs consist of concentric shells of graphitic carbon and were initially reported by Ugarte in 1992 [3]. They exhibit extraordinary physical properties, that render them interesting for industrial applications, with tribology as a prominent example, in addition to biomedical and electronic uses [16]. To fully realize the practical applications of CNOs, an in-depth knowledge of potential environmental and human health hazards is necessary in order to enable a responsible and accurate risk assessment [17,18]. The aquatic environment deserves special attention in this regard, since spills or release during the manufacturing process, storage, transportation or application could lead to water contamination, thus their ecotoxicological risks need to be carefully evaluated [19]. Some nanoparticles could be uptaken by planktonic or sediment dwelling invertebrates and thus enter the food chain, potentially posing a hazard for wildlife and humans. The use of aquatic animals for ecotoxicological investigations is strongly indicated by the EU chemical safety policy REACH (Registration, Evaluation, Authorization and Restriction of Chemicals) [20], aiming to characterize by 2018 the impact on aquatic ecosystems of all chemical substances present in the European market above a set threshold level (1 metric ton per year). Crustaceans (Daphnia) and aquatic plants (algae) are selected for short-term toxicity and growth inhibition tests, respectively, while fish are used for the next annual tonnage level (>10 metric tons).

Toxicological data for CNOs is very scarce. For small CNOs with diameters of about 5 nm, just a few studies with living organisms have been reported to date. Recently, we examined the toxicological and inflammatory potential of CNOs *in vivo* on C57BL/6 wild-type mice [21]. In addition, several *in vitro* studies with a variety of cell lines have been performed [22,23]. These initial studies revealed a low cytotoxic as well as a low inflammatory potential for small, chemically functionalized CNOs. However, no information is available on water organisms, in contrast to work that has already been conducted with other carbon nanomaterials such as fullerenes and their derivatives [24,25,26,27], several nanoforms of graphene [28,29,30,31] and CNTs. A large number of different water organisms have been exposed to CNTs including algae [32,33,34], fish [35,36], *Daphnia* [37,38], and many others [39,40,41,42]. In general, they display significant, concentration dependent detrimental effects on the studied water organisms. In addition to acute toxicity, photo-toxicity also seems to be an important issue since illumination of carbon nanomaterials eventually leads to the generation of reactive oxygen species [27,43,44,45]. In the case of CNTs, contamination with heavy metal nanoparticles (typically cobalt, molybdenum or iron), which act as catalysts during the CNT synthesis, deserve special attention and might increase the observed toxic effects dramatically. Complex purification strategies have been developed in order to facilitate the complete removal of catalyst particles from CNT samples [46,47,48,49]. Another strategy is to include control experiments as described by Petersen for the toxicological impacts of the catalyst materials [50].

**Figure 1:**
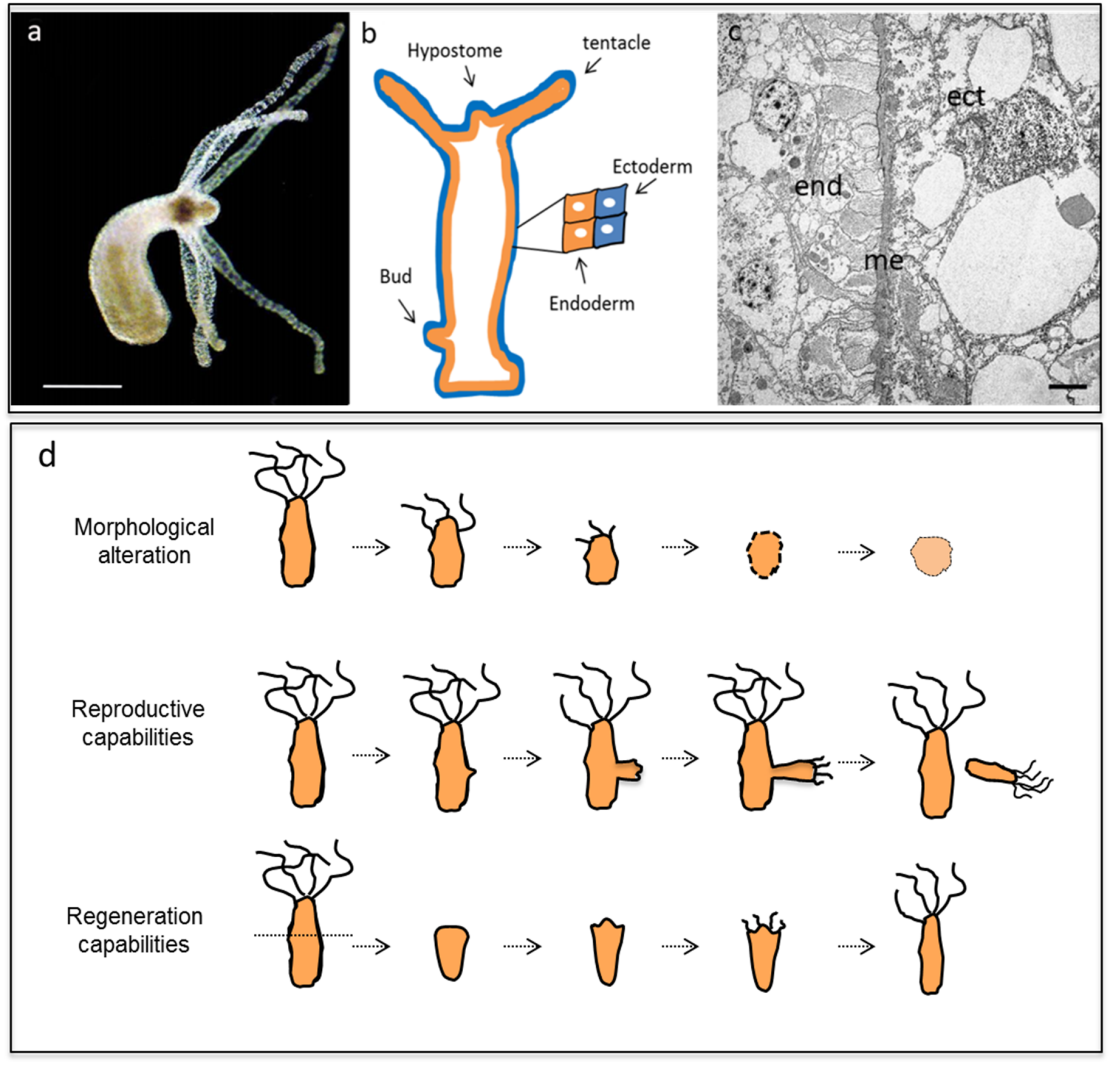
Structural anatomy of *Hydra vulgaris*. (**a**) The *Hydra* polyp is structured as a single hollow and transparent tube with a foot responsible for the anchoring to a substrate and a mouth surrounded by a crown of 6–8 tentacles. Scale bar, 500 µm. (**b**) Schematic representation of *Hydra* tissue organization with the inner endoderm (end) and an outer ectoderm (ect) separated by an acellular matrix, the mesoglea (me). The same structure is shown by transmission electron microscopy (**c**) of tissue cross sections. Scale bar, 10 µm. **(d**) Developmental potential of *Hydra vulgaris.*

In the present study, we investigate for the first time the toxicological effects of small CNOs with different substitution patterns on the freshwater polyp *Hydra vulgaris*, which is a commonly used model organism for developmental and environmental studies (Figure 1) [51,52,53]. *Hydra vulgaris* belongs to the phylum Cnidaria, and thus represents a very basal animal from an evolutionary perspective. It is widely used in biology to investigate, describe and manipulate important phenomena such as development, regeneration and differentiation [54]. Although very simple, the animal is indeed composed of two epithelial cell layers (an inner endoderm and an outer ectoderm (Figure 1) with some specialized cell types and a nerve net able to control and regulate many physiological functions [55]. *Hydra* has also been used in the past to study the toxicity of effluents and heavy metals [56,57]. Owing to its remarkable regenerative capacity, it was employed to examine the teratogenic potential of several chemicals including ethinylestradiol, bisphenol A, nonylphenol and several pharmaceuticals [58,59,60]. Furthermore, the molecular tools available, *i.e.*, whole genomic sequence, gain and loss of function techniques, may enable us to study at the molecular level the mechanisms underlying the toxicity. *Hydra* offers several potential advantages in probing the toxic effects of nanomaterials due to its small size, simple body architecture, asexual reproduction mode, tissue transparency and the availability of reliable protocols that enable toxicological evaluations at the whole animal, cell and molecular levels. Over the last years several nanoparticles have been tested for toxicity on *Hydra*. The results obtained allowed the identification of the pivotal role of not only the chemical composition of the inorganic core, but also of the size, shape and chemical coating of the nanoparticle surfaces [61,62,63]. PEG-coated CdSe/CdS quantum rods at the typical concentrations employed for biological imaging applications did not exhibit adverse effects on animal health, making them useful as probes for long-term cell tracking [64]. On the other hand, ultrasmall CdTe quantum dots (QDs) induced several alterations on *Hydra* morphology, resulting in the progressive disintegration of tentacle and body tissue, impairment of reproductive and regenerative capabilities, induction of apoptotic pathways and modulation of gene expression profiles similar to those observed with cadmium salts [65,66]. Silica nanoparticles induced different effects, impacting behavior and tissue organization [67], while gold and iron oxide nanoparticles did not exert toxic effects even at high concentrations [68,69]. This observation highlights the importance of adequate protection of the nanoparticle core by chemical coatings to prevent the cytoplasmic release of potential hazardous ions. While for metal-based nanoparticles the toxicity may arise from the component metals, in the case of nanomaterials composed of non-metallic elements, unexpected responses can be observed, possibly due to nanoscale dependent effects governing the interaction between the nanoparticle and the cell membrane. In this regard, the evaluation of possible toxicity effects caused by CNOs when interacting with a eukaryote aquatic organism is important, before designing CNO-based biodevices for biological or environmental applications.

The presence of toxic compounds in the medium bathing living polyps, or inside animal tissues may affect animal survival and physiology at various levels, from induction of severe damages at tissue/cell level, to impairment of asexual reproductive capability or regeneration. (Image used with permission from [51]). Reliable assays have been developed to quantify the effects of a given compound on these phenomena, taking place over short and long periods.

The investigated CNOs were either pristine CNOs (***p-CNO***) [70] or chemically modified CNOs. The surfaces of the CNOs were decorated by the radical addition of aromatic diazonium salts, following the so-called *Tour* reaction [16,21,22,23,71] which was described earlier for the surface modification of CNTs [72,73]. The functionalities examined herein include benzoic acid (***benz-CNO)***, pyridine (***py-CNO***) and methylpyridinium iodide (***py+-CNO***). The CNO nanomaterials were characterized by thermogravimetric analysis (TGA), dynamic light scattering (DLS), Z-potential, Fourier transform infrared (FT-IR) and Raman spectroscopy. Their effects on *Hydra* were subsequently investigated *in vivo* by monitoring and quantifying the morphology, reproductive and regenerative capabilities, and *in vitro* by evaluating cell apoptosis.

## 2. Results and Discussion

**Figure 2:**
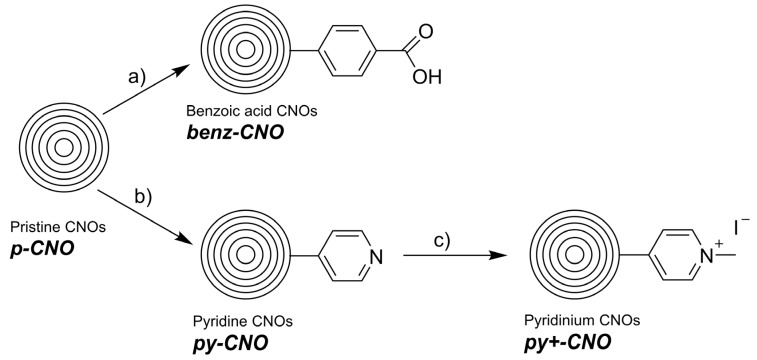
Scheme of synthetic procedures for the surface functionalization of pristine CNOs. (**a**) 4-Aminobenzoic acid, NaNO_2_, HCl, dimethylformamide/water; (**b**) 4-Aminopyridine, NaNO_2_, 4 N HCl/dimethylformamide. (**c**) Iodomethane, acetonitrile. All CNOs contain multiple functionalities on the surface; this scheme is simplified.

**Figure 3:**
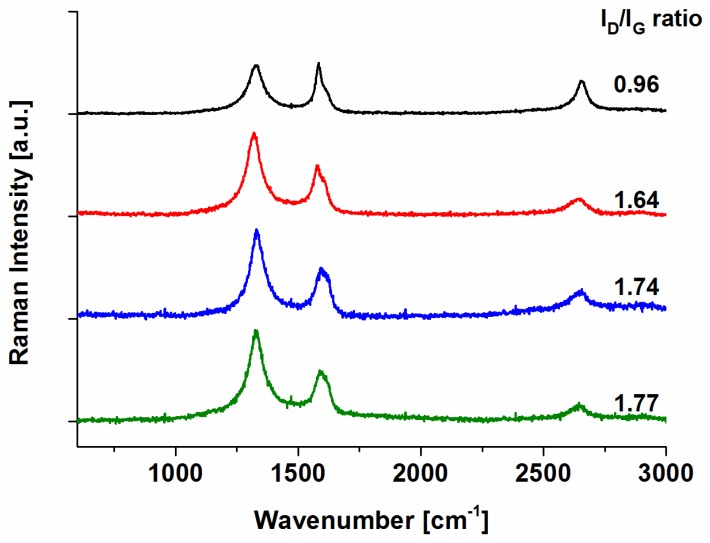
Raman spectra of ***p-CNO*** (black line), ***benz-CNO*** (red line), ***py-CNO*** (blue line) and ***py+-CNO*** (green line).

The benzoic functionalized and the pyridine functionalized CNOs were synthesized following the so-called Tour-reaction from *in situ* prepared diazonium salts of 4-aminobenzoic acid or 4-aminopyridine, respectively, and ***p-CNO***. The pyridinium functionalized CNO derivative (***py+-CNO*)** was prepared by reacting ***py-CNO*** with iodomethane (Figure 2). All reactions led to functionalized CNO nanomaterials as black powders, which were characterized by TGA, DLS and Z-potential, FT-IR and Raman spectroscopies. Atomic Force Microscopy and Transmission Electron Microscopy studies were previously conducted to get the size distribution of the CNOs. The presented data unambiguously revealed a size of about 5 nm in diameter (over 100 individual CNOs were analyzed) [23]. Raman spectra of functionalized CNOs display an enhancement of the D-band (1320 cm^−1^) compared to the G-band (1580 cm^−1^), confirming the functionalization of the CNO’s graphitic structure [15]. The I_D_/I_G_ ratio (Raman intensity of the D-band *vs.* the G-band) increases from a value of 0.96 for ***p-CNO*** to values of 1.64 and 1.74 for ***benz-CNO*** and ***py-CNO*** respectively (Figure 3). As expected the methylation of the pyridine moiety of the ***py-CNO***, leading to ***py+CNO***, did not alter the I_D_/I_G_ ratio significantly. FT-IR spectra of all CNOs are presented in Figure S1. While ***p-CNO*** did not show any notable IR absorption bands, despite the broad plasmonic CNO absorption over the whole spectral area, the surface functionalization of the CNOs revealed weak but notable IR bands. However, the strong background absorption of the CNOs resulted in weakly established IR absorption features, making it difficult to clearly identify specific functional groups. Thermogravimetric analysis (TGA) confirmed the successful functionalizations. All functionalized CNOs show a decrease in the decomposition temperature and an increase in weight loss at 400 ^°^C compared to pristine CNOs (Table S1, Figure S2). In order to explore the effect of the functionalization on the surface charge of the CNOs, the Z-potentials were determined. The Z-potentials were found to be −39.9 mV (±4.4 mV) for ***benz-CNO*** and −31.1 mV (±4.7 mV) for ***py-CNO***. The methylation of the pyridine moiety had a significant effect on the Z-potential which was found to be −2.9 mV (±5.9 mV) for ***py+-CNO****s*, confirming the formation of pyridinium cations. The observed dispersibility for the negatively charged ***benz-CNO*** and ***py-CNO*** was significantly better than for ***py+-CNO***. The almost neutral surface charge of ***py+-CNO*** led to an increased tendency to form aggregates. Dynamic light scattering (DLS) experiments corroborated these findings. While ***benz-CNO****s* and ***py-CNO****s* were found to have average hydrodynamic radii between 450 and 550 nm, when being dispersed in phosphate buffered saline (PBS), ***py+-CNO****s* revealed larger hydrodynamic radii of about 750 nm. When determining the hydrodynamic radii of the CNO agglomerates in *Hydra* medium, smaller agglomerates are observed for ***benz-CNO****s*. ***benz-CNO****s* revealed much smaller values at around 250 nm, while ***py-CNO****s* showed hydrodynamic radii at around 500 nm, relatively similar to the values observed in the PBS buffer. ***Py+-CNO****s*, however, showed slightly larger hydrodynamic radii in the Hydra medium with values at around 800 nm. A rational explanation for the observed differences is the low concentration of dissolved salts in the *Hydra* medium, compared to PBS, next to a different pH. This aspect, together with the different surface modifications of the CNO nanoparticles is of great importance for the behavior of CNOs dispersed in any aqueous medium, thus rendering our approach of studying and comparing differently functionalized CNOs of high significance.

The general tendency of the CNOs to aggregate with time makes precise determinations of the particle size in solution difficult. To summarize, the physico-chemical characterization of the different CNO nanomaterials clearly reveals changes of the CNO surface due to chemical functionalization. The formation of CNO agglomerates was observed, a significant finding for the interpretation of the findings in the *in vivo* studies. Subsequently, the effects of these differently functionalized CNOs on *Hydra* were investigated.

### 2.1. Hydra Exposure to CNO: Impact on Morphology and Survival Rate

In contrast to higher metazoans, where patterning mechanisms and cell fate determination generally occur only during embryogenesis, in *Hydra* the tissues are in a state of constant growth and tissue replacement [74]. Exposure to a toxicant may immediately impair the cell and tissue physiology. This unique sensitivity to pollutants enables the assessment of toxic effects of any medium-suspended compound by monitoring and quantitatively estimating several parameters by following standardized protocols, both *in vivo* and *in vitro,* such as measurements of morphological traits, reproductive and regenerative efficiencies (Figure 1d), and the assessment of apoptotic nuclei rates.

**Figure 4:**
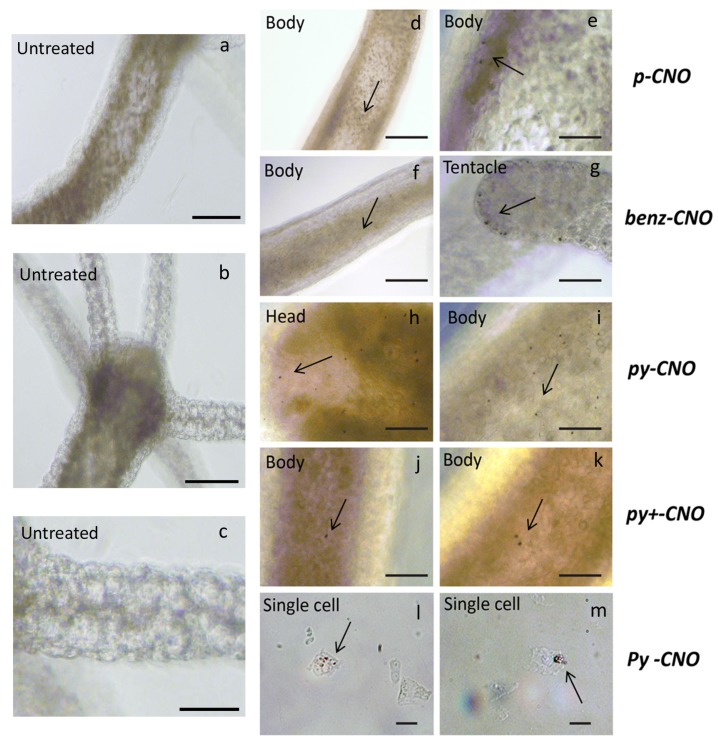
*In vivo* uptake and biodistribution of CNO nanoparticles in *Hydra*. (**a**–**c**) Bright field images of a living untreated polyp. Scale bar: 200 µm. (**d**–**m**) All different types of CNOs were internalized into *Hydra* tissues, and images show small differences in the uptake efficiency. Internalized CNOs appear as black granular structures and are present in the tentacles, heads and body columns. Scale bars: 500 µm in (**f**); 50 µm in (**g**); 200 µm in **(e**,**h**–**k**). Single cell suspensions obtained from treated animals show a granular structure within the cytoplasm of epithelial cells, suggesting a macropynocitosis mechanism mediating the entrance of large amounts of CNOs into the cells. Representative single cells obtained by animals treated with ***py-CNO*** are shown. Scale bar: 20 µm in (**l**,**m**).

To assess CNO toxicity, groups of 25 animals were exposed to different concentrations of CNOs, ranging from 0.01 mg/mL up to 0.1 mg/mL. No internalization was detectable at the lowest dose tested for all CNO nanoparticles (data not shown), and the animals did not show morphological alterations induced by the presence of CNO, even at the highest concentration tested. In the presence of toxicants, *Hydra* polyps may be induced to several aberrant reactions, starting from contractions of limited or extended body regions (tentacles, body column or both), to aberrant behaviors, such as body paralysis or tentacle writhing, up to induction of programmed cell death or necrosis, depending on the stressor. Even when exposing *Hydra* polyps to CNO at the highest concentrations possible (dictated by the sample initial concentration and by its stability in the *Hydra* medium) we did not observed and behavioral or morphological alterations. The results are shown in Figure S3, where a numerical score, previously developed by Wilby [75] was employed to quantify tissue damage induced by a variety of toxicants. No significant differences were observed between CNO treated polyps (0.1 mg/mL) and controls. To avoid any problem possibly related to aggregation, further toxicological evaluations were performed using the low dose 0.05 mg/mL. Each type of CNO nanoparticle was added to the culture medium bathing living polyps, monitored under a stereomicroscope following 24 h of incubation. The images of treated animals showed comparable levels of internalization for all CNO types, into the ectodermal and endodermal layers (Figure 4), confirming previous studies of dynamic processes occurring between the two cell epithelia, causing migration of labeled cells or free nanoparticles from the ectoderm to the endoderm [65]. CNO aggreagates are visible in the body, head and tentacles of animals as small dark spots (Figure 4d–k). By dissociating treated animals into single cell suspensions, the granular structures are clearly evident within the cytoplasm, likely representing storage or lysosomal vesicles, mediating the accumulation or the degradation of the internalized material, respectively (Figure 4l,m). These results are similar to those obtained with other nanoparticles, both fluorescent [65] and not [68]. Over recent years the mechanism of internalization of several nanoparticles into *Hydra* was investigated. In the case of gold nanoparticles ultrastructural analysis by electron microscopy was performed to dissect the pathways of the nanoparticles from the initial interaction with the *Hydra* membrane to exocytosis [69]. Several mechanisms were identified as driving both the uptake and the secretion, integrating and supporting those previously achieved using fluorescent QDs [65] These are independent from the chemical composition and surface net charge which dictate the initial interaction between the nanoparticle and cell membranes and then the efficiency of internalization, a common mechanism (micropynocitosis) mediates the uptake of medium suspended nano and microparticles. Due to the microsized vesicles observed in the animals treated with CNOs, we suggest a similar mechanism for the uptake and accumulation of CNOs, not excluding the possible aggregation of CNOs in the *Hydra* medium, which would result in an identical pattern of uptake. A slightly lower rate of internalization was observed for ***p-CNO*** and ***py+-CNO***, as shown by the bright field images in Figure 4, where only a few dark spots are visible (Figure 4d,e,j,k). However, compared to other nanoparticles, *i.e.*, quantum dots, quantum rods, gold or iron oxide nanoparticles, the overall efficiency of internalization by macropinocytosis was similar for all CNO types.

### 2.2. Hydra Exposure to CNO: Impact on Head Regeneration and Reproductive Capabilities

**Figure 5:**
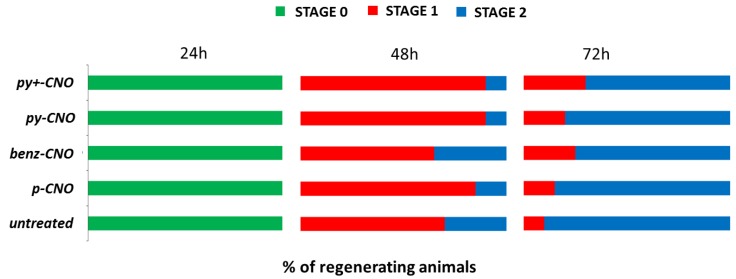
Effect of CNOs on the regenerative potential of *Hydra*. Groups of 25 polyps were bisected in the upper gastric region and incubated in the presence of 0.05 mg/mL of CNO. The regenerating polyps, monitored through a stereomicroscope, were grouped into three stages according to their tentacle morphogenetic process [54]. Nanoparticle treatment did not impair the regenerative potential of *Hydra* since no differences were observed in the percentage of regenerating animals between treated and untreated polyps, relative to each stage. The graph is representative of three independent biological replicate (total number of polyps: 75). No significant differences were found between treatments.

**Figure 6:**
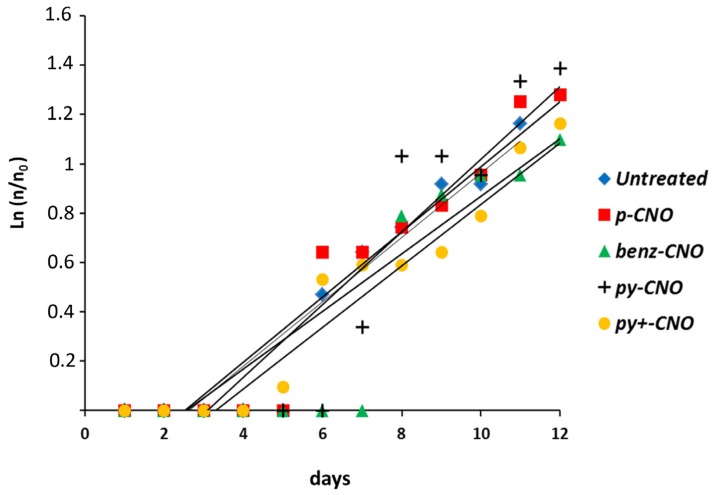
Effect of CNOs on the asexual reproductive potential of *Hydra.* Five *Hydra* founders, untreated or incubated for 24 h with 0.05 mg/mL of CNOs, were washed out and monitored every day for bud growth and detachment. The graph shows *n*/*n_0_* values at each time point. No significant differences in the growth rate between treated and untreated animals was observed, indicating the absence of CNO toxicity on a long term scale. By comparing linear regression slopes (ANOVA two-way test; *p* < 0.005), no significant differences were found between growth rates of *Hydra* populations exposed to CNOs.

As a long-term toxicity criterion, the regenerative and reproductive capabilities of polyps treated with CNOs were estimated. *Hydra* polyps easily regenerate amputated body parts. Over the first 48 h post-amputation (p.a.) morphogenetic processes take place, followed by cell proliferation to restore the adult size. Groups of 25 polyps were bisected in the upper gastric region and incubated in the presence of different doses of CNO nanoparticles. The polyps were monitored through a stereomicroscope and were grouped in three stages according to their tentacle morphogenetic process: stage zero indicates the complete inhibition of regeneration (zero tentacles); stage 1, indicates heads with aberrant tentacles (one or two), while stage 2 indicates normal regeneration (from four to six tentacles) [62,66,75]. No differences were detected between the regeneration efficiency of treated and untreated polyps, as shown in Figure 5, indicating that the presence of CNOs in *Hydra* tissues does not impair the regenerative potential. The reproductive capability of CNO treated polyps was also measured. In *Hydra*, the epithelial cells structuring its body continuously divide and migrate towards the animal ends, leading to the formation of new individuals, budding from the gastric region, and detaching from the mother in about three days. Due to this asexual reproduction modality, the population growth rate is an indirect measure of the *Hydra* tissue growth rate and cell viability, and it is routinely used as a toxicity endpoint. A group of five founder animals (n_0_) either untreated or incubated for 24 h with 0.05 mg/mL of each type of CNOs were daily fed and monitored for bud formation and detachment over two weeks. The total number of individuals (n) used to calculate the growth rate constant (k) over the duration of the experiment (t) was defined by the equation ln(n/n_0_) = kt. The obtained k values were compared to those obtained for the untreated animals. No significant differences were observed between the growth rates of treated and untreated animals, showing the absence of toxic effects of these nanoparticles over a long time scale (Figure 6).

### 2.3. Hydra Exposure to CNO: Impact on Apoptosis

We evaluated the effects of CNO exposure on apoptosis by monitoring nuclear morphology by fluorescence microscopy. The process through which a cell undergoes during programmed death is called apoptosis and is characterized by a precise sequence of events including nuclear condensation, activation and cleavage of caspases, finally leading then to cell death. Apoptosis is a physiological phenomenon that is used by metazoans to regulate the number of cells in growing tissues and it turns out to be a key developmental program employed by *Hydra* polyps to control cell proliferation in response to feeding, regeneration and non-self cell removal. The remarkable similarity observed for the apoptotic cells and the overall apoptosis mechanism (caspases involved, positive and negative regulators) between *Hydra* and higher metazoans shows how conserved and evolutionarily important this mechanism is [76].

**Figure 7:**
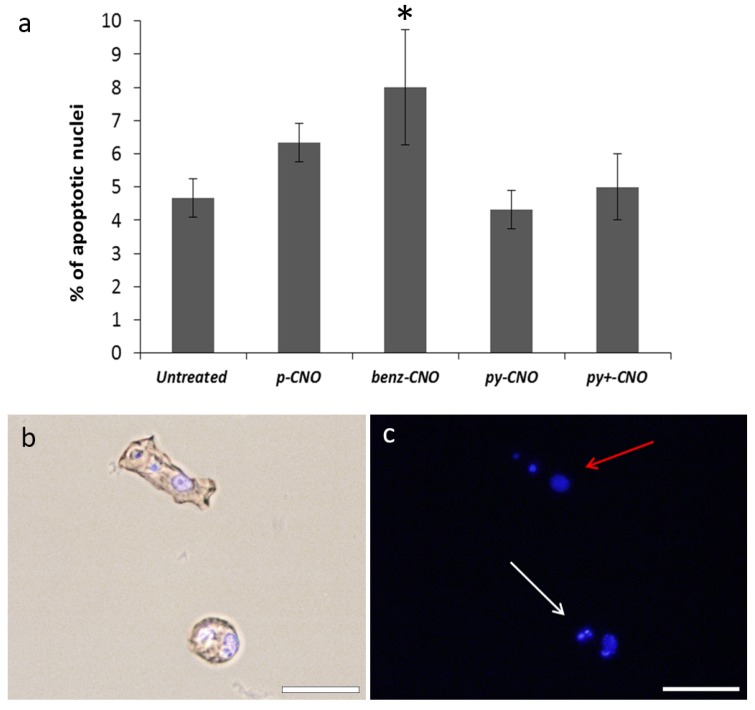
Cellular assessment of apoptosis induction by CNO nanoparticles. Following 24 h incubation in 0.05 mg/mL CNO dispersions, polyps were macerated into single cells and the percentage of apoptotic nuclei was determined by counting the DAPI-stained fragmented nuclei. (**a**) The graph represents the percentage of apoptotic nuclei in normal and treated conditions. ***Benz-CNO*** treatment induces a significant increase of apoptotic nuclei (the asterisk denotes statistical significance according to a one-way ANOVA analysis followed by Tukey’s multiple comparison post-test, *p* < 0.001). (**b**) Fluorescence microscopy imaging of single cells prepared from CNO-treated *Hydra*. The morphology observed by phase contrast indicates an ectodermal epithelial cell. Scale bar: 20 µm. **(c**) Fluorescence imaging following DAPI staining shows a normal nucleus (red arrow) and a typical apoptotic pyknotic nucleus (white arrow). Scale bar: 20 µm.

To evaluate the amount of apoptotic cells, *Hydra* treated for 24 h with 0.05 mg/mL CNO dispersions were macerated into a single-cell suspension and nuclei counter-stained with DAPI. The number of pyknotic nuclei, reflecting an apoptotic effect, was counted. Figure 7 illustrates that only ***benz-CNO*** induced a slight increase of the apoptosis rate of *Hydra* cells, while all other CNO derivatives gave results comparable to those for the untreated control. Overall, our results lead us to the conclusion that no notable negative effects of dispersed, functionalized CNOs on *Hydra* were observed.

## 3. Conclusions

Despite the wide arrays developed to assess nanomaterials toxicity, the overall absence of adverse effects induced by CNOs on short and long term toxicity in *Hydra* suggest a reasonable degree of biosafety of this new class of materials. Despite the scarcity of data on CNO toxicity, our results are in line with part of the current literature which promotes carbon-based nanostructures (e.g., fullerenes) as safe materials in several biological models, such as bacteria [77], fungi [78], human cells [79], *Drosophila* [80] and mice [81]. Some aqueous dispersions of fullerenes induced adverse effects in aquatic invertebrates, which were primarily correlated with their preparation techniques [82], or to the high dose. Noteworthy, all the toxicity data have been obtained by using different types of fullerenes (varying in size, surface chemistry, solubility, aggregation/agglomeration) and experimental setups (exposure time, biological models). This may suggest that before drawing final conclusions on the toxicological impact of carbon-based nanomaterials, large-scale analysis, designed to dissect the influence of each nanostructure property, must be considered. In addition, current studies do not provide sufficient information on long-term exposures, which would definitely shed light on fullerenes nanosafety. In conclusion, nanoecotoxicologists should keep in mind that a complete assessment of the environmental risk is a complex issue, where multiple environmental players determine the stability, the uptake and the fate of particulate matter by aquatic plant/animal species. The understanding of such intricate dynamics requires multidisciplinary skills and remains difficult to be carried out by a single laboratory. Thus in the next future systems biology and bioinformatics tools may be included to assist not only the analysis of large datasets, but also in the prediction of potential harmful effects in the designing of new nanoparticle based products.

## 4. Experimental Section

### 4.1. Nanomaterial Synthesis

All starting materials, reagents and solvents were purchased from commercial suppliers (Sigma-Aldrich, St. Louis, MS, USA or Fisher, Waltham, MA, USA) in high-purity and used without further purification. *Pristine CNOs* (***p-CNO***) were synthesized following a previously published procedure [71].

#### 4.1.1. *benz-CNO*

Sodium nitrite (1.47 g/21.3 mmol) was dissolved in 20 mL of deionized (DI) water and cooled to 0 °C. This solution was added to a solution of 4-aminobenzoic acid (2.88 g/21.0 mmol) in 30 mL of DMF at 0 °C Two hundred microliters of concentrated-HCl were added as the mixture was stirred for 30 min at 0 °C. *Pristine CNOs* (30 mg) were dispersed in 20 mL DMF by ultrasonication for 20 min and the dispersion was added to the reaction mixture, which was stirred at 0 °C for 4h and at RT for an additional 3 days. Following this, the CNOs were separated from the reaction mixture by centrifugation (30 min, 2100 g) and purified by subsequent redispersion-centrifugation steps in DI water, DMF, and methanol. After drying at 60 °C overnight, 25 mg of ***benz-CNO*** were recovered.

#### 4.1.2. *py-CNO*

Sodium nitrite (2.94 g/42.6 mmol) was dissolved in 5 mL deionized water and subsequently added dropwise at 0 °C to a solution of 4-aminopyridine (3.96 g/42 mmol) in 4 N HCl (30 mL). The mixture was stirred for 30 min, then ***p-CNO*** (60 mg), dispersed in 30 mL of DMF after 10 min of ultrasonication, were added. After stirring for 4 h at 0 °C, the reaction mixture was stirred for three days at room temperature. The CNO nanomaterials were recovered and purified by means of centrifugation. The sample was centrifuged at 2100 g for 30 min, followed by removal of the supernatant and redispersion in DMF. Another centrifugation-redispersion cycle in DMF was performed, followed by a final cycle in methanol. Then, the CNOs were dried at 65 °C for two days and 56 mg of ***py-CNO*** were recovered.

#### 4.1.3. *py+-CNO*

10 mg of ***py-CNO*** were dispersed in 8 mL of acetonitrile by ultrasonication for 10 min, then iodomethane (1.0 mL) was added. The mixture was stirred for 3 days and the solvents were then removed under high-vacuum. 11 mg of a black powder, consisting of ***py+-CNO***, were recovered.

### 4.2. Nanomaterial Characterization

#### 4.2.1. FT-IR Spectroscopy

FT-IR spectroscopic studies were carried out on a Bruker Vertex 70v FT-IR spectrometer (Bruker, Ettlingen, Germany) equipped with a Platinum ATR accessory.

#### 4.2.2. Raman Spectroscopy

Raman spectra were measured on a 800 UV LabRam Raman microscope (Horiba Jobin Yvon, Longjumeau, France). For the Raman measurements, the samples were excited with a built-in 632 nm laser. The samples were deposited by adding the dry compound to a drop of methanol on the glass slide. The slides were dried in air for two hours.

#### 4.2.3. Dynamic Light Scattering (DLS) and Zeta-Potential

DLS measurements were performed on the Malvern Nano-ZS (Worcestershire, UK) instrument operating in backscattering (173°) mode and analyzed with the proprietary software Zetasizer, with automatic selection of the optimal detector position and number of independent measurements. PBS pH 7.4 was chosen to mimic biological conditions and to ensure a pH stable environment. In addition *Hydra* medium (see below) was used for DLS experiments. CNO samples were weighted (about 1.0 mg) and dispersed in DI water to a final concentration of 1.0 mg/mL and sonicated for 30 min at 37 kHz. The dispersions were then diluted in PBS or *Hydra* medium, respectively to achieve a final concentration of CNOs of about 10 μg/mL. The suspension was then sonicated at 37 kHz for additional 5 min and particle sizes were measured instantaneously. Z-potential measurements were performed with the same apparatus using disposable proprietary Z-potential cuvettes. Dilutions of the CNO samples were prepared in a low ionic strength phosphate buffer (0.01 M, pH 7.4) to a final concentration of 10 μg/mL and sonicated (37 kHz, 5 min) prior to measurements.

#### 4.2.4. Thermogravimetric Analysis

TGA was conducted on a TA Q500 analyzer (TA Instruments, New Castle, DE, USA), using a Pt pan as sample holder. The measurement was performed in air using a heating rate of 10 °C/min, after equilibrating the sample at 30 °C for 5 min and then at 100 °C for an additional 20 min, the sample weight was monitored until 900 °C.

### 4.3. Biological Methods

#### 4.3.1. Animal Culturing and *in vivo* Experiments

*Hydra vulgaris* (strain Zurich, originally obtained by P. Tardent, Zurich, Switzerland), were cultured in *Hydra* medium (1 mM CaCl_2_, 0.1 mM NaHCO_3_, pH 7.0), fed on alternate days with *Artemia nauplii* at 18 °C with 12:12 h dark/light cycles. Polyps from homogeneous populations, three weeks old, were selected for experiments, performed at 18 °C. The tests were initiated by collecting groups of 20 animals in plastic multiwells, followed by the addition of 0.05 mg/mL CNO (***p-CNO***, ***benz-CNO***, ***py-CNO*** and ***py+-CNO***) in 300 μL of *Hydra* medium to each well and incubation for 24 h. Nanoparticle uptake was monitored *in vivo* by a stereomicroscope (Olympus ZSXRFL2, Tokyo, Japan). Following extensive washes, *in vivo* imaging was accomplished by an inverted microscope (Axiovert 100, Zeiss, Oberkochen, Germany) equipped with a digital colour camera (Olympus, DP70). For imaging acquisition and analysis the software system Cell F (Olympus) was used.

#### 4.3.2. Hydra Cell and Tissue Analysis

*Hydra* polyps were incubated in maceration solution (1:1:13 acetic acid, glycerol, water) to obtain a single cell suspension and fixed in formaldehyde. Whole *Hydra* polyps were anaesthetized in 2% urethane in *Hydra* medium for 2 min. The relaxed and elongated polyps were fixed with Lavdowsky’s fixative (ethanol/formaldehyde/acetic acid/water at 50:10:4:40), rehydrated, and mounted on microscope slides in 50% glycerol in PBS (8 g/L NaCl; 0.2 g/L KCl; 1.44 g/L Na_2_HPO_4_·7H_2_O; 0.24 g/L KH_2_PO_4_).

#### 4.3.3. Hydra Growth Rates and Regeneration

Animals (five polyps with one bud) were treated with 0.05 mg/mL of CNOs for 24 h, washed and placed in 3.5 cm Petri dishes (1 animal/dish). Control polyps at the same developmental stage were not treated. Both treated and untreated *Hydra* were fed once daily for 14 days. The growth rate constant (k) of an exponentially growing group of animals is defined as ln(n/n_0_) = kt where n is the number of animals at time t and n_0_ the number of founder animals. Two independent experiments were conducted for each growth rate.

For regeneration experiments, groups of 25 polyps were bisected in the upper gastric region and incubated in the presence of nanoparticles. The regenerating polyps, monitored through a stereomicroscope, were grouped in three stages according to their tentacle morphogenetic process. Three independent biological replicates were performed.

### 4.4. Assessment of Apoptosis

Apoptotic cell death was evaluated by 4'-6-Diamidino-2-phenylindole (DAPI) staining. Briefly, untreated and CNO treated polyps (5 animals for each treatment) were macerated and the single cell suspension was fixed with 4% paraformaldehyde and spread on slides. After extensive washing in PBS, macerates were stained with DAPI for 2 min and washed in PBS. Slides were observed with phase-contrast fluorescent microscopy to detect pyknotic nuclei. More than 300 cells were counted for each treatment and the percentage of apoptotic nuclei was determined. At least two slides were inspected in each experiment and the percentage of apoptotic nuclei was determined. Three biological replicates were carried out for each CNO treatment

### 4.5. Statistical Analysis

Median scores of morphological condition were compared by nonparametric Friedman analysis. A *t*-test (*p* < 0.001) was used to test for significance between treatments. The slope of the regression curves obtained from single population growth rate was tested for significance using a two-way ANOVA (*p* < 0.001). In the case of apoptosis assessment, one-way ANOVA analysis followed by Tukey’s multiple comparison post-test (*p* < 0.001) was employed to test statistical significance.

## Supplementary Materials

Supplementary materials can be accessed at: http://www.mdpi.com/2079-4991/5/3/1331/s1.
