# Supplementary material for: Impact of Carbon Nano-Onions on Hydra vulgaris as a Model Organism for Nanoecotoxicology
Source: Nanomaterials (Basel). 2015 Aug 13;5(3):1331–50. doi: 10.3390/nano5031331 (PMC5304644; doi:10.3390/nano5031331)
Supplement: Supplementary file 1 [file nanomaterials-05-01331-s001.pdf]

Supplementary Materials

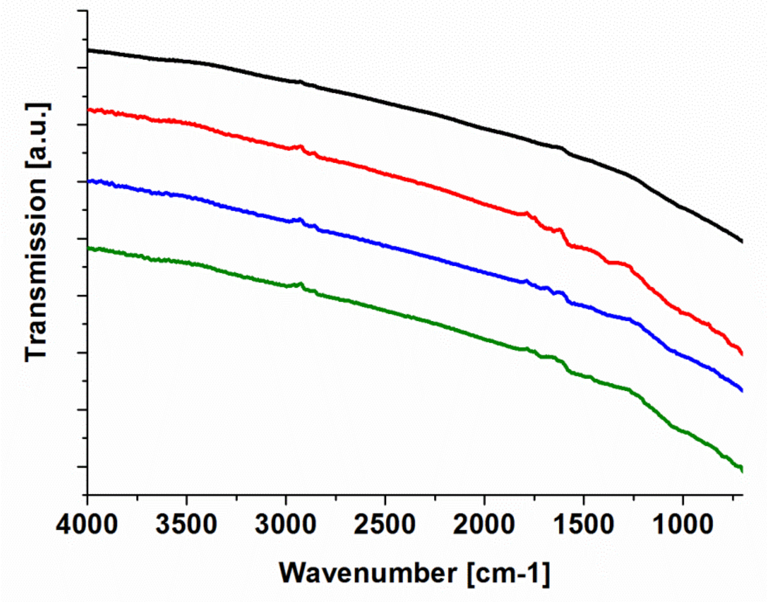

Figure S1. FT-IR spectra of ***p-CNO*** (black line), ***benz-CNO*** (red line), ***py-CNO*** (blue line), and ***py+-CNO*** (green line).

Table S1. Thermogravimetric analysis (TGA) data for the CNOs.

Sample	Weight Loss at 400 °C	Decomposition Temperature	Residue
pCNO	0.9%	595 °C	5.75%
benz-CNO	10.9%	570 °C	5.59%
py-CNO	5.0%	556 °C	5.52%
py+-CNO	13.0%	564 °C	3.80%

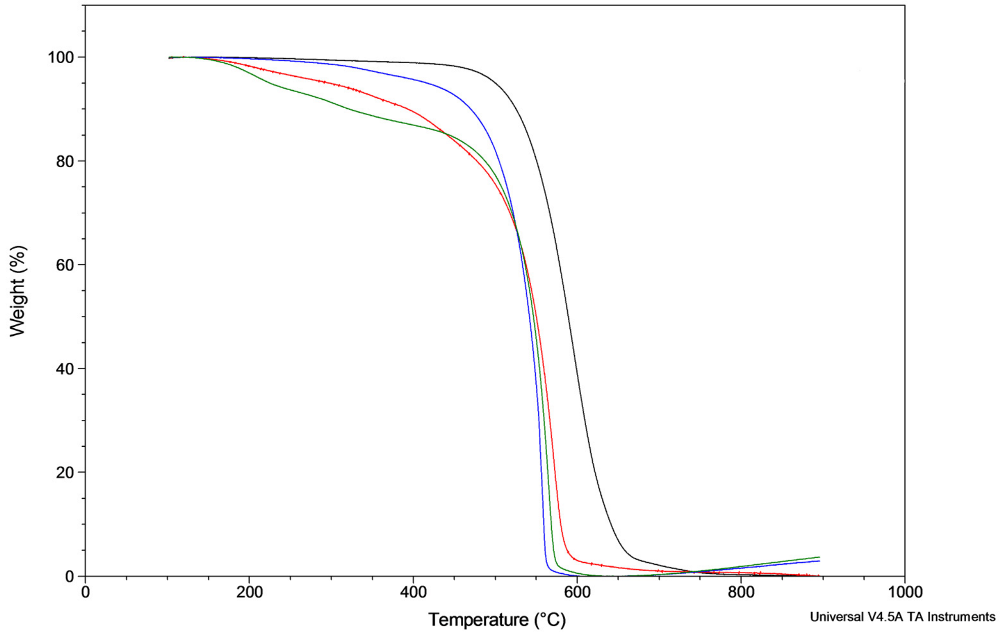

Figure S2. TGA curves of ***p-CNO*** (black line), ***benz-CNO*** (red line), ***py-CNO*** (blue line), and ***py+-CNO*** (green line).

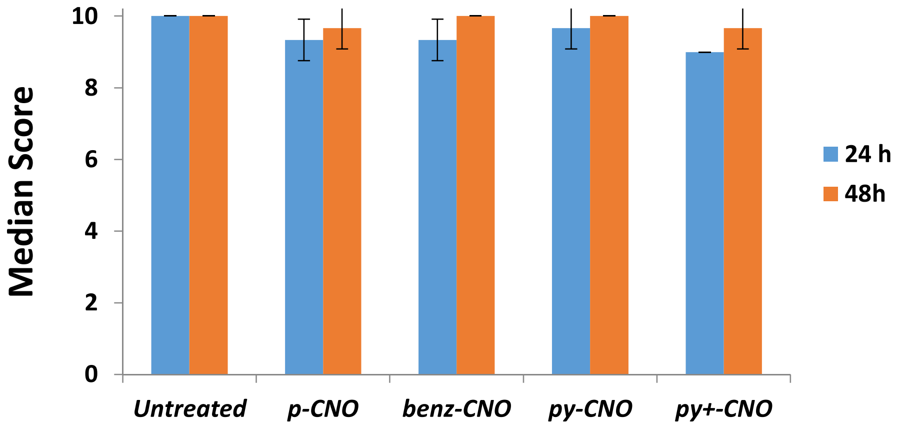

Figure S3. Evaluation of potential morphological aberrations induced by different CNO types on *Hydra vulgaris* polyps.

Toxicity tests were carried out on groups of 20 polyps exposed to 0.1 mg/mL of each CNO type, microscopically inspected at 24 h intervals. A numerical score was assigned ranging from 10 for a normal polyp to 0 if it was disintegrated, as originally described by Wilby [75] and previously adapted by our group. The median scores were reported for each CNO type. Treated animals did not present significant morphological alterations, as shown by the median value of the numerical scores, ranging from 9 to 10, and thus indicating polyp health. Experiments were performed in triplicate.

© 2015 by the authors; licensee MDPI, Basel, Switzerland. This article is an open access article distributed under the terms and conditions of the Creative Commons Attribution license (http://creativecommons.org/licenses/by/4.0/).
